# Supplementary material for: Impact of Infliximab Biosimilar CT-P13 Dose and Infusion Interval on Real-World Drug Survival and Effectiveness in Patients with Ankylosing Spondylitis
Source: J Clin Med. 2021 Oct 1;10(19):4568. doi: 10.3390/jcm10194568 (PMC8509762; doi:10.3390/jcm10194568)
Supplement: Supplementary file 1 [file jcm-10-04568-s001.zip › jcm-1359106-supplementary.pdf]

Supplementary materials

Impact of infliximab biosimilar CT-P13 dose and infusion interval on real-world drug survival and effectiveness in patients with ankylosing spondylitis

Shin-Seok Lee, Tae-Hwan Kim, Won Park, Yeong-Wook Song, Chang-Hee Suh, Soo-Kyoung Kim and Dae-Hyun Yoo

Correspondence to:

Professor Dae-Hyun Yoo
Department of Rheumatology, Hanyang University Hospital for Rheumatic Diseases, 222-1 Wangsimni-ro, Seongdong-gu, Seoul 04763, Republic of Korea and Hanyang University Institute for Rheumatology Research, 222-1 Wangsimni-ro, Seongdong-gu, Seoul 04763, Republic of Korea
Email: dhyoo@hanyang.ac.kr

Table S1. Baseline demographics and disease characteristics by baseline dose group (naïve patients).

	Overall (n = 175 [1])	Baseline dose			p-value [2]
		<4 mg/kg (n = 42)	≥4–<5 mg/kg (n = 84)	≥5 mg/kg (n = 49)	
Sex, n (%)					
Female	48 (27.4)	14 (33.3)	18 (21.4)	16 (32.7)	0.2315 [3]
Male	127 (72.6)	28 (66.7)	66 (78.6)	33 (67.3)	
Median (IQR) treatment duration, months	20.3 (7.40–35.2)	15.2 (5.77–28.5)	22.5 (7.80–35.1)	24.2 (5.30–44.4)	0.5398
Median (IQR) disease duration, years	1 (0–5)	2 (0–5)	1 (0–5)	1 (1–4)	0.6058
Median (IQR) age, years	36 (28–49)	42 (30–52)	33 (26–44)	38 (32–50)	0.0481
Body weight, kg					
n	169	39	83	47	
Median (IQR)	67.0 (60.0–75.0)	73.0 (58.0–80.0)	68.0 (62.0–73.0)	60.0 (58.0–76.0)	0.0527
BMI, kg/m^2					
n	134	35	56	43	
Median (IQR)	23.06 (21.05–25.88)	24.22 (22.44–27.15)	22.78 (21.08–24.00)	22.94 (20.70–26.16)	0.1699
BASDAI score					
n	140	30	67	43	
Median (IQR)	7.48 (6.55–8.33)	7.00 (6.09–8.00)	7.50 (6.80–8.30)	7.64 (6.50–9.10)	0.0781
ESR, mm/h					
n	168	40	82	46	
Median (IQR)	34 (14–65)	43 (18–67)	35 (18–70)	31 (9–51)	0.2982
CRP, mg/L					
n	168	40	82	46	
Median (IQR)	1.39 (0.62–3.75)	2.03 (0.74–3.23)	1.36 (0.68–4.44)	1.17 (0.32–3.20)	0.2212

[1] Baseline dose was missing for 44 patients because of missing weight information (n = 34) or missing dose after induction period in naïve patients (n = 10). [2] p-values were determined by Kruskal–Wallis test, unless otherwise specified. [3] p-value was determined by chi-squared test. BASDAI, Bath Ankylosing Spondylitis Disease Activity Index; BMI, body mass index; CRP, C-reactive protein; ESR, erythrocyte sedimentation rate; IQR, interquartile range.

Table S2. Baseline demographics and disease characteristics by baseline dose group (switched patients).

	Overall (*n* = 95 [1])	Baseline dose <4 mg/kg (*n* = 29)	≥4–<5 mg/kg (*n* = 33)	≥5 mg/kg (*n* = 33)	*p*-value [2]
Sex, *n* (%)					
Female	20 (21.1)	6 (20.7)	6 (18.2)	8 (24.2)	0.8320 [3]
Male	75 (78.9)	23 (79.3)	27 (81.8)	25 (75.8)	
Median (IQR) treatment duration, months	31.0 (9.80–49.4)	42.1 (17.7–49.2)	25.6 (7.47–48.3)	31.0 (7.50–51.9)	0.6165
Median (IQR) disease duration, years	5 (2–9)	5 (4–8)	7 (3–9)	5 (2–7)	0.5453
Median (IQR) age, years	40 (31–52)	45 (38–56)	34 (30–43)	42 (33–52)	0.0287
Body weight, kg					
n	95	29	33	33	
Median (IQR)	66.7 (59.0–73.0)	64.0 (58.1–77.0)	67.2 (61.8–72.0)	65.9 (56.8–73.0)	0.7345 [4]
BMI, kg/m^2					
n	89	28	29	32	
Median (IQR)	23.53 (21.87–25.35)	23.80 (22.73–25.37)	23.45 (21.13–25.86)	23.10 (20.61–25.02)	0.5634 [4]
BASDAI score					
n	72	25	21	26	
Median (IQR)	1.91 (0.58–4.08)	0.57 (0.30–1.66)	1.01 (0.66–2.80)	4.24 (2.86–5.10)	<0.0001
ESR, mm/h					
n	91	29	31	31	
Median (IQR)	8 (5–19)	13 (5–42)	8 (5–16)	6 (4–17)	0.1431
CRP, mg/L					
n	91	29	31	31	
Median (IQR)	0.14 (0.05–0.80)	0.27 (0.05–0.80)	0.24 (0.04–0.80)	0.10 (0.05–0.43)	0.6867

[1] Baseline dose was missing for 23 patients because of missing weight information. [2] *p*-values were determined by Kruskal–Wallis test, unless otherwise specified. [3] *p*-value was determined by chi-squared test. [4] *p*-value was determined by one-way analysis of variance. BASDAI, Bath Ankylosing Spondylitis Disease Activity Index; BMI, body mass index; CRP, C-reactive protein; ESR, erythrocyte sedimentation rate; IQR, interquartile range.

Table S3. Baseline demographics and disease characteristics by dose analysis group (overall).

	Overall (n = 235 [1])	Dose analysis group			p-value [2]
		Constant dose (n = 217)	Increased dose (n = 12)	Decreased dose (n = 6)	
Sex, n (%)					
Female	57 (24.3)	167 (77.0)	6 (50.0)	5 (83.3)	0.1158 [3]
Male	178 (75.7)	50 (23.0)	6 (50.0)	1 (16.7)	
Median (IQR) treatment duration, months	27.7 (12.6–47.8)	26.4 (12.5–47.8)	43.7 (19.8–49.6)	33.5 (32.6–44.4)	0.4002
Median (IQR) disease duration, years	3 (1–7)	3 (1–7)	2 (1–6)	3 (1–5)	0.9088
Median (IQR) age, years	38 (29–50)	36 (29–50)	47 (33–62)	37 (26–63)	0.2623
Baseline dose group, n (%)					
n	235	217	12	6	
<4 mg/kg	59 (25.1)	50 (23.0)	9 (75.0)	0	N/C
≥4–<5 mg/kg	101 (43.0)	96 (44.2)	3 (25.0)	2 (33.3)	
≥5 mg/kg	75 (31.9)	71 (32.7)	0	4 (66.7)	
Body weight, kg					
n	231	213	12	6	
Median (IQR)	66.7 (59–74)	66.9 (60–75)	63.0 (58–68)	70.1 (60–73)	0.4311
BMI, kg/m^2					
n	190	172	12	6	
Median (IQR)	23.44 (21.63–25.48)	23.41 (21.35–25.52)	23.71 (23.02–25.18)	22.76 (20.82–26.13)	0.6893
BASDAI score					
n	187	172	10	5	
Median (IQR)	6.70 (3.83–7.90)	6.75 (3.83–8.00)	4.84 (0.50–6.50)	6.50 (6.20–6.71)	0.1175
ESR, mm/h					
n	225	208	12	5	
Median (IQR)	21 (7–50)	20 (7–48)	24 (12–64)	78 (50–78)	0.0276
CRP, mg/L					
n	225	208	12	5	
Median (IQR)	0.80 (0.22–2.64)	0.80 (0.16–2.54)	0.84 (0.30–2.57)	4.45 (1.60–5.46)	0.1533

[1] Dose was missing for 102 patients because of missing weight information or not meeting the minimum number of infusions for dose change analysis. [2] p-values were determined by Kruskal–Wallis test, unless otherwise specified. [3] p-value was determined by Fisher's exact test. BASDAI, Bath Ankylosing Spondylitis Disease Activity Index; BMI, body mass index; CRP, C-reactive protein; ESR, erythrocyte sedimentation rate; IQR, interquartile range; N/C, not calculable.

Table S4. Baseline demographics and disease characteristics by dose analysis group (naïve patients).

	Overall (n = 152 [1])	Dose analysis group			p-value [2]
		Constant dose (n = 142)	Increased dose (n = 5)	Decreased dose (n = 5)	
Sex, n (%)					
Female	42 (27.6)	38 (26.8)	2 (40.0)	4 (80.0)	0.2792 [3]
Male	110 (72.4)	104 (73.2)	3 (60.0)	1 (20.0)	
Median (IQR) treatment duration, months	25.0 (11.5–37.0)	24.4 (11.5–36.9)	13.1 (9.97–26.6)	33.6 (33.4–44.4)	0.0981
Median (IQR) disease duration, years	1 (0–5)	1 (0–5)	1 (0–1)	2 (1–3)	0.4048
Median (IQR) age, years	34 (27–49)	34 (27–48)	47 (27–61)	32 (26–63)	0.6740
Baseline dose group, n (%)					
n	152	142	5	5	
<4 mg/kg	33 (21.7)	30 (21.1)	3 (60.0)	0	N/C
≥4–<5 mg/kg	74 (48.7)	71 (50.0)	2 (40.0)	1 (20.0)	
≥5 mg/kg	45 (29.6)	41 (28.9)	0	4 (80.0)	
Body weight, kg					
n	148	138	5	5	
Median (IQR)	67.1 (59.3–75.0)	67.1 (60.0–75.0)	60.0 (54.0–68.0)	68.2 (60.0–73.0)	0.5256
BMI, kg/m^2					
n	113	103	5	5	
Median (IQR)	23.26 (21.10–25.70)	23.36 (21.10–25.88)	23.62 (22.99–23.68)	22.58 (20.82–22.94)	0.8332
BASDAI score					
n	124	115	4	5	
Median (IQR)	7.48 (6.55–8.40)	7.60 (6.60–8.50)	6.13 (5.61–7.43)	6.50 (6.20–6.71)	0.0529
ESR, mm/h					
n	145	135	5	5	
Median (IQR)	33 (13–67)	32 (12–60)	25 (18–73)	78 (50–78)	0.1076
CRP, mg/L					
n	145	135	5	5	
Median (IQR)	1.40 (0.63–3.70)	1.39 (0.62–3.60)	2.86 (0.87–3.06)	4.45 (1.60–5.46)	0.4775

[1] Dose was missing for 67 patients because of missing weight information or not meeting the minimum number of infusions for dose change analysis. [2] p-values were determined by Kruskal–Wallis test, unless otherwise specified. [3] p-value was determined by Fisher's exact test. BASDAI, Bath Ankylosing Spondylitis Disease Activity Index; BMI, body mass index; CRP, C-reactive protein; ESR, erythrocyte sedimentation rate; IQR, interquartile range; N/C, not calculable.

Table S5. Baseline demographics and disease characteristics by dose analysis group (switched patients).

	**Overall (*n* = 83 [1])**	**Dose analysis group**			***p*-value [2]**
		**Constant dose (*n* = 75)**	**Increased dose (*n* = 7)**	**Decreased dose (*n* = 1)**	
Sex, *n* (%)					
Female	15 (18.1)	12 (16.0)	3 (42.9)	0	0.2715 [3]
Male	68 (81.9)	63 (84.0)	4 (57.1)	1 (100.0)	
Median (IQR) treatment duration, months	39.2 (16.7–50.2)	36.6 (16.6–50.7)	49.2 (42.1–50.2)	5.77 (N/C)	0.1258
Median (IQR) disease duration, years	6 (3–9)	6 (3–9)	5 (2–11)	15 (N/C)	0.2452
Median (IQR) age, years	41 (32–53)	40 (32–53)	46 (39–63)	41 (N/C)	0.5857
Baseline dose group, *n* (%)					
n	83	75	7	1	
<4 mg/kg	26 (31.3)	20 (26.7)	6 (85.7)	0	N/C
≥4–<5 mg/kg	27 (32.5)	25 (33.3)	1 (14.3)	1 (100.0)	
≥5 mg/kg	30 (36.1)	30 (40.0)	0	0	
Body weight, kg					
n	83	75	7	1	
Median (IQR)	66.0 (59.0–73.0)	66.0 (59.0–73.0)	64.0 (58.1–67.2)	72.0 (N/C)	0.6631
BMI, kg/m^2					
n	77	69	7	1	
Median (IQR)	23.53 (21.94–25.35)	23.45 (21.64–25.15)	24.89 (23.05–28.20)	26.13 (N/C)	0.1925
BASDAI score					
n	63	57	6	0	
Median (IQR)	1.87 (0.51–3.88)	1.94 (0.62–3.83)	1.08 (0.30–4.20)	N/C	0.5504 [4]
ESR, mm/h					
n	80	73	7	0	
Median (IQR)	8 (5–21)	8 (5–18)	22 (8–59)	N/C	0.1046 [4]
CRP, mg/L					
n	80	73	7	0	
Median (IQR)	0.21 (0.05–0.80)	0.14 (0.05–0.80)	0.80 (0.20–1.76)	N/C	0.0944 [4]

[1] Dose was missing for 35 patients because of missing weight information or not meeting the minimum number of infusions for dose change analysis. [2] *p*-values were determined by Kruskal–Wallis test, unless otherwise specified. [3] *p*-value was determined by Fisher's exact test. [4] *p*-value was determined by two-sided Wilcoxon's rank sum test using a normal approximation. BASDAI, Bath Ankylosing Spondylitis Disease Activity Index; BMI, body mass index; CRP, C-reactive protein; ESR, erythrocyte sedimentation rate; IQR, interquartile range; N/C, not calculable.

Table S6. Median (IQR) numbers of annual infusions overall and for naïve and switched patients.

	Year 1	Year 2	Year 3	Year 4	Year 5
			Overall		
n	327	216	155	100	64
Median (IQR)	6.0 (5.0–7.0)	6.0 (4.0–6.0)	6.0 (4.0–6.0)	6.0 (5.0–6.0)	2.0 (1.0–3.0)
			Naïve		
n	209	132	89	44	24
Median (IQR)	7.0 (5.0–7.0)	6.0 (4.0–6.5)	5.0 (3.0–6.0)	6.0 (4.0–7.0)	2.0 (2.0–3.0)
			Switched		
n	118	84	66	56	40
Median (IQR)	6.0 (5.0–7.0)	6.0 (5.0–6.0)	6.0 (4.0–6.0)	6.0 (5.0–6.0)	1.0 (1.0–2.5)

IQR, interquartile range.

Table S7. Baseline demographics and disease characteristics by interval analysis group (overall).

	Overall (n = 224 [1])	Interval analysis group			p-value [2]
		Constant interval (n = 114)	Increased interval (n = 79)	Decreased interval (n = 31)	
Sex, n (%)					
Female	53 (23.7)	26 (22.8)	19 (24.1)	8 (25.8)	0.9363 [3]
Male	171 (76.3)	88 (77.2)	60 (75.9)	23 (74.2)	
Median (IQR) treatment duration, months	33.4 (21.5–49.4)	32.7 (17.0–50.1)	33.4 (25.0–47.8)	35.3 (21.5–50.9)	0.9108
Median (IQR) disease duration, years	3 (1–7)	3 (1–8)	2 (1–5)	5 (1–7)	0.0420
Median (IQR) age, years	38 (30–49)	40 (32–49)	33 (27–49)	40 (29–49)	0.1715
Baseline dose group, n (%)					
n	186	94	63	29	
<4 mg/kg	52 (28.0)	26 (27.7)	19 (30.2)	7 (24.1)	0.2032 [3]
≥4–<5 mg/kg	80 (43.0)	34 (36.2)	30 (47.6)	16 (55.2)	
≥5 mg/kg	54 (29.0)	34 (36.2)	14 (22.2)	6 (20.7)	
Body weight, kg					
n	183	94	60	29	
Median (IQR)	67.0 (60.0–74.0)	67.1 (60.0–75.0)	68.0 (60.0–73.0)	63.4 (58.2–73.0)	0.7172 [4]
BMI, kg/m^2					
n	144	81	41	22	
Median (IQR)	23.50 (21.68–25.33)	23.67 (21.83–26.57)	23.38 (21.72–24.89)	22.93 (21.41–24.22)	0.1625 [4]
BASDAI score					
n	186	96	67	23	
Median (IQR)	6.50 (2.20–7.70)	6.15 (1.95–7.76)	6.71 (4.50–7.90)	5.47 (0.98–7.30)	0.1755
ESR, mm/h					
n	218	110	77	31	
Median (IQR)	23 (8–51)	21 (7–48)	30 (13–51)	18 (7–68)	0.1372
CRP, mg/L					
n	218	110	77	31	
Median (IQR)	0.80 (0.24–2.50)	0.80 (0.14–1.77)	1.30 (0.66–3.56)	0.59 (0.11–3.13)	0.0137

[1] Interval was missing for 113 patients because of a missing infusion date record or not meeting the minimum number of infusions for interval change analysis. [2] p-values were determined by Kruskal–Wallis test, unless otherwise specified. [3] p-value was determined by chi-squared test. [4] p-value was determined by one-way analysis of variance. BASDAI, Bath Ankylosing Spondylitis Disease Activity Index; BMI, body mass index; CRP, C-reactive protein; ESR, erythrocyte sedimentation rate; IQR, interquartile range.

Table S8. Baseline demographics and disease characteristics by interval analysis group (naïve patients).

	**Overall (*n* = 140** [1]**)**	**Interval analysis group**			***p*-value** [2]
		**Constant interval (*n* = 61)**	**Increased interval (*n* = 63)**	**Decreased interval (*n* = 16)**	
Sex, *n* (%)					
Female	36 (25.7)	15 (24.6)	16 (25.4)	5 (31.3)	0.8606 [3]
Male	104 (74.3)	46 (75.4)	47 (74.6)	11 (68.8)	
Median (IQR) treatment duration, months	29.2 (17.3–41.9)	25.4 (14.9–43.5)	32.4 (24.3–37.0)	35.3 (20.1–52.0)	0.0938
Median (IQR) disease duration, years	1 (0–5)	1 (0–5)	1 (0–5)	2 (1–4)	0.9988
Median (IQR) age, years	34 (28–48)	35 (28–47)	32 (27–47)	41 (31–49)	0.3183
Baseline dose group, *n* (%)					
n	118	51	51	16	
<4 mg/kg	29 (24.6)	12 (23.5)	12 (23.5)	5 (31.3)	0.7582 [4]
≥4–<5 mg/kg	56 (47.5)	22 (43.1)	27 (52.9)	7 (43.8)	
≥5 mg/kg	33 (28.0)	17 (33.3)	12 (23.5)	4 (25.0)	
Body weight, kg					
n	115	51	48	16	
Median (IQR)	68.0 (60.0–75.0)	70.0 (60.0–76.4)	68.0 (60.0–74.5)	66.3 (58.1–74.0)	0.8698 [5]
BMI, kg/m^2					
n	82	43	30	9	
Median (IQR)	23.53 (21.29–25.71)	23.73 (20.82–26.93)	22.59 (21.64–25.30)	22.91 (21.80–23.68)	0.3625
BASDAI score					
n	118	53	53	12	
Median (IQR)	7.43 (6.60–8.30)	7.64 (6.70–8.89)	7.40 (6.50–8.09)	7.13 (6.70–7.60)	0.2317 [5]
ESR, mm/h					
n	136	59	61	16	
Median (IQR)	34 (15–67)	30 (18–60)	35 (15–62)	44 (10–74)	0.7535
CRP, mg/L					
n	136	59	61	16	
Median (IQR)	1.46 (0.77–3.68)	1.39 (0.64–3.57)	1.60 (0.80–4.00)	1.87 (0.47–4.20)	0.5367

[1] Interval was missing for 79 patients because of a missing infusion date record or not meeting the minimum number of infusions for interval change analysis. [2] *p*-values were determined by Kruskal–Wallis test, unless otherwise specified. [3] *p*-value was determined by chi-squared test. [4] *p*-value was determined by Fisher's exact test. [5] *p*-value was determined by one-way analysis of variance. BASDAI, Bath Ankylosing Spondylitis Disease Activity Index; BMI, body mass index; CRP, C-reactive protein; ESR, erythrocyte sedimentation rate; IQR, interquartile range.

Table S9. Baseline demographics and disease characteristics by interval analysis group (switched patients).

	**Overall** (*n* = 84 [1])	**Interval analysis group**			***p*-value** [2]
		**Constant interval** (*n* = 53)	**Increased interval** (*n* = 16)	**Decreased interval** (*n* = 15)	
Sex, *n* (%)					
Female	17 (20.4)	11 (20.8)	3 (18.8)	3 (20.0)	1.0000 [3]
Male	67 (79.8)	42 (79.2)	13 (81.3)	12 (80.0)	
Median (IQR) treatment duration, months	48.4 (30.1–50.6)	48.9 (30.3–50.9)	48.5 (33.8–49.8)	47.2 (22.0–50.7)	0.5006
Median (IQR) disease duration, years	6 (3–9)	6 (3–9)	5 (3–7)	7 (6–12)	0.0693
Median (IQR) age, years	41 (33–53)	41 (35–52)	44 (28–58)	40 (29–52)	0.5724 [4]
Baseline dose group, *n* (%)					
n	68	43	12	13	
<4 mg/kg	23 (33.8)	14 (32.6)	7 (58.3)	2 (15.4)	0.0348 [3]
≥4–<5 mg/kg	24 (35.3)	12 (27.9)	3 (25.0)	9 (69.2)	
≥5 mg/kg	21 (30.9)	17 (39.5)	2 (16.7)	2 (15.4)	
Body weight, kg					
n	68	43	12	13	
Median (IQR)	64.6 (59.1–71.0)	65.9 (61.0–73.0)	63.9 (57.3–70.0)	62.6 (61.0–66.6)	0.6705 [4]
BMI, kg/m^2					
n	62	38	11	13	
Median (IQR)	23.46 (22.01–25.21)	23.33 (22.01–26.31)	23.66 (22.27–24.25)	22.96 (21.41–24.45)	0.7490
BASDAI score					
n	68	43	14	11	
Median (IQR)	1.05 (0.33–2.89)	1.66 (0.50–3.40)	0.42 (0.20–1.10)	0.98 (0.30–3.57)	0.0883
ESR, mm/h					
n	82	51	16	15	
Median (IQR)	12 (5–29)	8 (5–23)	15 (7–41)	17 (5–29)	0.4353
CRP, mg/L					
n	82	51	16	15	
Median (IQR)	0.18 (0.05–0.80)	0.20 (0.05–0.80)	0.17 (0.04–0.57)	0.12 (0.03–0.80)	0.7863

[1] Interval was missing for 34 patients because of a missing infusion date record or not meeting the minimum number of infusions for interval change analysis. [2] *p*-values were determined by Kruskal–Wallis test, unless otherwise specified. [3] *p*-value was determined by chi-squared test. [4] *p*-value was determined by one-way analysis of variance. BASDAI, Bath Ankylosing Spondylitis Disease Activity Index; BMI, body mass index; CRP, C-reactive protein; ESR, erythrocyte sedimentation rate; IQR, interquartile range.

Table S10. Baseline demographics and disease characteristics by combined analysis group (overall).

	**Overall** (***n*** **= 186)**	**Combined analysis group**		***p*-value** [1]
		**Combined constant** (***n*** **= 85)**	**Combined changed** (***n*** **= 101)**	
Sex, *n* (%)				
Female	46 (24.7)	18 (21.2)	28 (27.7)	0.3026 [2]
Male	140 (75.3)	67 (78.8)	73 (72.3)	
Median (min–max) treatment duration, months	33.1 (9.97–56.8)	31.4 (11.2–56.8)	33.8 (9.97–54.9)	0.6559
Median (min–max) disease duration, years	3 (0–33)	4 (0–14)	3 (0–33)	0.0776
Median (min–max) age, years	37 (18–75)	40 (19–72)	34 (18–75)	0.1093
Baseline dose group, *n* (%)				
<4 mg/kg	52 (28.0)	21 (24.7)	31 (30.7)	0.1217 [2]
≥4–<5 mg/kg	80 (43.0)	33 (38.8)	47 (46.5)	
≥5 mg/kg	54 (29.0)	31 (36.5)	23 (22.8)	
Body weight, kg				
n	183	85	98	
Median (min–max)	67.0 (41.0–102.0)	67.0 (41.0–102.0)	66.0 (46.0–90.0)	0.7982 [3]
BMI, kg/m^2				
n	144	72	72	
Median (min–max)	23.50 (16.48–32.93)	23.70 (16.48–32.93)	23.32 (17.59–32.18)	0.2079 [3]
BASDAI score				
n	151	68	83	
Median (min–max)	6.70 (0.00–10.00)	6.37 (0.00–10.00)	6.70 (0.10–10.00)	0.8195
ESR, mm/h				
n	180	81	99	
Median (min–max)	24 (0–126)	20 (1–126)	30 (0–124)	0.0122
CRP, mg/L				
n	180	81	99	
Median (min–max)	0.80 (0.01–15.00)	0.80 (0.01–14.70)	1.10 (0.01–15.00)	0.0413

[1] *p*-values were determined by Wilcoxon's rank sum test, unless otherwise specified. [2] *p*-value was determined by chi-squared test. [3] *p*-value was determined by two-sample t-test. BASDAI, Bath Ankylosing Spondylitis Disease Activity Index; BMI, body mass index; CRP, C-reactive protein; ESR, erythrocyte sedimentation rate; max, maximum; min, minimum.

Table S11. Baseline demographics and disease characteristics by combined analysis group (naïve patients).

	Overall (n = 118)	Combined analysis group		p-value [1]
		Combined constant (n = 46)	Combined changed (n = 72)	
Sex, n (%)				
Female	31 (26.3)	10 (21.7)	21 (29.2)	0.3713 [2]
Male	87 (73.7)	36 (78.3)	51 (70.8)	
Median (min–max) treatment duration, months	29.4 (9.97–54.9)	25.9 (11.4–54.2)	32.6 (9.97–54.9)	0.0945
Median (min–max) disease duration, years	1 (0–14)	2 (0–12)	1 (0–14)	0.8549
Median (min–max) age, years	34 (18–74)	36 (21–71)	33 (18–74)	0.3696
Baseline dose group, n (%)				
<4 mg/kg	29 (24.6)	10 (21.7)	19 (26.4)	0.8133 [2]
≥4–<5 mg/kg	56 (47.5)	22 (47.8)	34 (47.2)	
≥5 mg/kg	33 (28.0)	14 (30.4)	19 (26.4)	
Body weight, kg				
n	115	46	69	
Median (min–max)	68.0 (41.0–102.0)	70.1 (41.0–102.0)	68.0 (46.0–90.0)	0.7775 [3]
BMI, kg/m^2				
n	82	38	44	
Median (min–max)	23.53 (17.51–32.93)	23.89 (17.51–32.93)	22.92 (17.59–32.18)	0.1603 [3]
BASDAI score				
n	99	39	60	
Median (min–max)	7.50 (4.10–10.00)	7.80 (4.10–10.00)	7.30 (4.11–10.00)	0.0228 [3]
ESR, mm/h				
n	114	44	70	
Median (min–max)	35 (2–126)	30 (2–126)	43 (2–124)	0.1852
CRP, mg/L				
n	114	44	70	
Median (min–max)	1.46 (0.01–15.00)	1.33 (0.03–14.70)	1.61 (0.01–15.00)	0.1713

[1] p-values were determined by Wilcoxon's rank sum test, unless otherwise specified. [2] p-value was determined by chi-squared test. [3] p-value was determined by two-sample t-test. BASDAI, Bath Ankylosing Spondylitis Disease Activity Index; BMI, body mass index; CRP, C-reactive protein; ESR, erythrocyte sedimentation rate; max, maximum; min, minimum.

Table S12. Baseline demographics and disease characteristics by combined analysis group (switched patients).

	**Overall (*n* = 68)**	**Combined analysis group**		***p*-value** [1]
		**Combined constant (*n* = 39)**	**Combined changed (n = 29)**	
Sex, *n* (%)				
Female	15 (22.1)	8 (20.5)	7 (24.1)	0.7214 [2]
Male	53 (77.9)	31 (79.5)	22 (75.9)	
Median (min–max) treatment duration, months	48.0 (11.2–56.8)	48.3 (11.2–56.8)	47.8 (12.4–52.6)	0.6553
Median (min–max) disease duration, years	6 (0–33)	7 (1–14)	6 (0–33)	0.5839
Median (min–max) age, years	41 (19–75)	40 (19–72)	43 (21–75)	0.7482 [3]
Baseline dose group, *n* (%)				
<4 mg/kg	23 (33.8)	11 (28.2)	12 (41.4)	0.0312 [2]
≥4–<5 mg/kg	24 (35.3)	11 (28.2)	13 (44.8)	
≥5 mg/kg	21 (30.9)	17 (43.6)	4 (13.8)	
Body weight, kg				
n	68	39	29	
Median (min–max)	64.6 (41.5–93.6)	65.9 (41.5–93.6)	63.4 (46–90)	0.5223 [3]
BMI, kg/m²				
n	62	34	28	
Median (min–max)	23.46 (16.48–31.20)	23.33 (16.48–31.20)	23.56 (17.97–30.68)	0.7550 [3]
BASDAI score				
n	52	29	23	
Median (min–max)	1.66 (0.00–9.16)	2.27 (0.00–9.16)	0.86 (0.10–7.00)	0.0753
ESR, mm/h				
n	66	37	29	
Median (min–max)	9 (0–90)	6 (1–90)	15 (0–80)	0.0811
CRP, mg/L				
n	66	37	29	
Median (min–max)	0.21 (0.01–4.69)	0.29 (0.01–1.93)	0.20 (0.01–4.69)	0.5125

[1] *p*-values were determined by Wilcoxon's rank sum test, unless otherwise specified. [2] *p*-value was determined by chi-squared test. [3] *p*-value was determined by two-sample t-test. BASDAI, Bath Ankylosing Spondylitis Disease Activity Index; BMI, body mass index; CRP, C-reactive protein; ESR, erythrocyte sedimentation rate; max, maximum; min, minimum.

Table S13. Median (IQR) BASDAI scores [1] by treatment group for combined analysis groups.

	Week 0	Week 54	Week 102	Week 156	Week 210
**Combined constant group**					
Overall					
n	72	73	57	42	31
Median (IQR)	6.32 (2.83–8.00)	2.60 (0.84–3.90)	2.40 (0.80–3.50)	2.55 (0.80–3.80)	2.60 (0.69–3.40)
Naïve					
n	39	39	26	18	12
Median (IQR)	7.80 (6.80–9.30)	2.20 (0.84–4.10)	1.75 (1.00–3.80)	1.90 (0.80–3.60)	1.44 (0.58–3.20)
Switched					
n	33	34	31	24	19
Median (IQR)	2.58 (0.90–3.80)	2.75 (0.84–3.40)	2.50 (0.80–3.30)	2.80 (0.72–3.90)	2.70 (0.69–4.00)
**Combined changed group**					
Overall					
n	87	85	76	53	35
Median (IQR)	6.70 (4.11–7.60)	1.32 (0.79–2.70)	1.19 (0.57–2.23)	1.20 (0.44–2.00)	1.00 (0.39–2.30)
Naïve					
n	63	60	54	34	18
Median (IQR)	7.30 (6.50–7.90)	1.83 (1.00–2.80)	1.35 (0.80–2.20)	1.31 (0.76–2.00)	1.35 (0.81–2.30)
Switched					
n	24	25	22	19	17
Median (IQR)	0.92 (0.27–2.85)	0.80 (0.40–1.54)	0.85 (0.40–2.26)	0.70 (0.29–2.00)	0.58 (0.18–1.55)

[1] BASDAI results are not presented for week 264 as there was only one evaluable patient. BASDAI, Bath Ankylosing Spondylitis Disease Activity Index; IQR, interquartile range.
